# δ-MnO_2_ nanoflower/graphite cathode for rechargeable aqueous zinc ion batteries

**DOI:** 10.1038/s41598-019-44915-8

**Published:** 2019-06-11

**Authors:** Sonti Khamsanga, Rojana Pornprasertsuk, Tetsu Yonezawa, Ahmad Azmin Mohamad, Soorathep Kheawhom

**Affiliations:** 1 Department of Chemical Engineering, Faculty of Engineering, Chulalongkorn University, Bangkok, 10330 Thailand; 2 Department of Materials Science, Faculty of Science, Chulalongkorn University, Bangkok, 10330 Thailand; 3 Research Unit of Advanced Materials for Energy Storage, Chulalongkorn University, Bangkok, 10330 Thailand; 4 Center of Excellence in Petrochemical and Materials Technology, Chulalongkorn University, Bangkok, 10330 Thailand; 5 Division of Materials Science and Engineering, Faculty of Engineering, Hokkaido University, Kita 13 Nishi 8, Sapporo, Hokkaido 060-8628 Japan; 6 School of Materials and Mineral Resources Engineering, Universiti of Sains Malaysia, 14300 Nibong Tebal, Pulau Pinang, Malaysia

**Keywords:** Batteries, Batteries

## Abstract

Manganese oxide (MnO_2_) is one of the most promising intercalation cathode materials for zinc ion batteries (ZIBs). Specifically, a layered type delta manganese dioxide (δ-MnO_2_) allows reversible insertion/extraction of Zn^2+^ ions and exhibits high storage capacity of Zn^2+^ ions. However, a poor conductivity of δ-MnO_2_, as well as other crystallographic forms, limits its potential applications. This study focuses on δ-MnO_2_ with nanoflower structure supported on graphite flake, namely MNG, for use as an intercalation host material of rechargeable aqueous ZIBs. Pristine δ-MnO_2_ nanoflowers and MNG were synthesized and examined using X-ray diffraction, electron spectroscopy, and electrochemical techniques. Also, performances of the batteries with the pristine δ-MnO_2_ nanoflowers and MNG cathodes were studied in CR2032 coin cells. MNG exhibits a fast insertion/extraction of Zn^2+^ ions with diffusion scheme and pseudocapacitive behavior. The battery using MNG cathode exhibited a high initial discharge capacity of 235 mAh/g at 200 mA/g specific current density compared to 130 mAh/g which is displayed by the pristine δ-MnO_2_ cathode at the same specific current density. MNG demonstrated superior electrical conductivity compared to the pristine δ-MnO_2_. The results obtained pave the way for improving the electrical conductivity of MnO_2_ by using graphite flake support. The graphite flake support significantly improved performances of ZIBs and made them attractive for use in a wide variety of energy applications.

## Introduction

Manganese dioxide (MnO_2_) is widely used as a cathode material in battery technologies because of its several advantageous properties such as low-cost, abundant, low toxicity, and environmental friendliness^1–3^. MnO_2_ were previously studied and applied for a variety of energy storage devices^4–7^. Also, it was applied in different metal-ion batteries including Li-ion battery (LIB)^8^, Mg-ion battery (MIB)^9^ and Zn-ion battery (ZIB)^10,11^. MnO_2_ cathodes are inexpensive and exhibit a high theoretical capacity. Recently, aqueous Zn/MnO_2_ batteries are considered as promising alternative energy devices due to their high safety and the abundance of both Zn, MnO_2_ and the electrolyte^12–14^.

However, MnO_2_ suffers from its poor conductivity that often occurs in high internal resistance of the electrode resulting in poor performance of the battery^15^. Therefore, to improve the performance of the MnO_2_ cathode, it is necessary to increase the specific surface area of MnO_2_ as well as the ion diffusion rate^16,17^. MnO_2_ has various crystallographic polymorphs such as α-MnO_2_, β-MnO_2_, and δ-MnO_2_, etc. Among these, δ-MnO_2_ was reported to be a potential intercalation host material for aqueous ZIBs^18^ due to a substantial interlayer distance for the reversible insertion/extraction of Zn^2+^ ions. δ-MnO_2_ can be prepared by a chemical reduction or hydrothermal process^19^. The conventional synthesis method is the direct reduction of KMnO_4_ aqueous solution by dropwise introduction of concentrated HCl. Previously, δ-MnO_2_ nano-flakes were synthesized and used in aqueous ZIBs^18^. It led to a significant increase in the power density of the ZIB. Another approach undertaken was to support MnO_2_ nanostructures on a matrix material with a high surface area^5,20,21^. It was observed that these nanostructures could accelerate the charge transport during the electrochemical redox process. In this respect, various carbonaceous materials, namely activated carbon, carbon nanotubes (CNTs), carbon nanofibers (CNFs), graphene and graphite, have been integrated with MnO_2_. MnO_2_/graphene nanoflowers were synthesized in the form of sandwich-structured nanoflowers which exhibited excellent super capacitive properties effectively making a very conductive electrode material for high-performance super capacitors^22^. However, it is significant that MnO_2_ supported on graphite has not been reported previously in ZIBs application. The crystal structure of graphite consists of parallel planes of carbon atoms which is conductive primarily along its planes^23,24^. In this way, graphite is classified as a semimetal due to its high electrical conductivity^25^. Therefore, MnO_2_ supported on graphite is considered as the candidate due to an improvement in electronic conductivity and an increase in the stability of the electrode materials for ZIBs.

The present study reports on δ-MnO_2_ nanoflower/graphite as a cathode host material for rechargeable aqueous ZIBs. The δ-MnO_2_ nanoflower supported on graphite not only increases the electrical conductivity and discharge capacity of the battery but also improves the insertion/extraction acceleration by increasing the active area of the δ-MnO_2_ nanoflower. Accordingly, the electrochemical properties and performances of the batteries which use the MNG as host material cathode are examined and discussed.

## Experimental

### Chemical and materials

Reagent grade chemicals were obtained and used without further purification unless noted otherwise. The graphite powder was purchased from Aldrich Company. Potassium permanganate (KMnO_4_), manganese sulfate monohydrate (MnSO_4_.H_2_O), zinc sulfate (ZnSO_4_), sulfuric acid (H_2_SO_4_), and cellulose acetate were purchased from Ajax Finechem. Nickel foam (0.5 mm thick, 100 PPI) was purchased from Qijing Trading Co., Ltd. Whatman filter paper No.1 was purchased from Sigma-Aldrich. Graphite foil was purchased from Shenzhen 3KS Electronic Material Co. Ltd. Zn sheet (99.99%) was purchased from Sirikul Engineering Ltd., Part.

### Preparation of δ-MnO_2_ nanoflower and δ-MnO_2_ nanoflower/graphite (MNG)

The pristine δ-MnO_2_ nanoparticles were synthesized by dissolving KMnO_4_ (1.98 g) in 60 mL of deionized (DI) water. Then, MnSO_4_.H_2_O (0.336 g) was dissolved in 20 mL of DI water. Next, the MnSO_4_.H_2_O solution was added dropwise to the KMnO_4_ solution, and continuous stirring followed for 30 min. Afterward, the mixture was transferred into a 100 mL Teflon autoclave and kept at 160 °C for 24 hr in an oil bath. The product was collected and washed with DI water several times. Then, it was dried at 80 °C for 12 hr. The MNG synthesis was similar to the method reported by Liu *et al*.^22^ with some modifications. Graphite (1.0 g) was mixed in DI water (500 mL) with KMnO_4_ (10.0 g). The mixture of graphite and KMnO_4_ was stirred for 18 hr. Then, 98% of H_2_SO_4_ (5 mL) was added dropwise into the mixture. The solution was continuously stirred for 1 hr and heated to and maintained at 80 °C for 1 hr. After that, the solution was diluted in 1 L of DI water and allowed to stand at room temperature. The solution was filtered using cellulose filter paper (pore size 11 µm). Then, the MNG precipitates were collected and washed by DI water several times until the violet color disappeared. Subsequently, the precipitates were dried at 80 °C overnight.

### Characterization and electrochemical measurement

X-ray Diffraction (XRD, Bruker AXS Model D8 Discover) of the powder samples was carried out with Cu Kα radiation at a scanning range of 5–80°. Field Emission Scanning Electron Microscope (FESEM, JEOL JSM-7610F, Tokyo, Japan) was used to take the morphology image and nanoflower size of MNG.

The cathode using MNG was prepared by mixing together 70% wt. of MNG, 20% wt. of carbon black (CB), and 10% wt. of cellulose acetate binder. Alternatively, the cathode using pristine δ-MnO_2_ was prepared by mixing together 70% wt. of the pristine δ-MnO_2_, 20% wt. of carbon black (CB), and 10% wt. of cellulose acetate binder. Acetone was used to adjust the viscosity of the slurries. Each mixed slurry was coated on graphite foil using a lab coating machine (AOT-FCM-250, AOT Electronic Technology Co., LTD) and dried at 70 °C under vacuum. The thickness of the cathode material deposited was 25 µm. The zinc anode was prepared by electrodeposition of zinc from ZnSO_4_ (0.5 M) aqueous solution onto Ni-foam using zinc sheet as a counter electrode at the current density of 60 mA/cm^2^. The amount of zinc deposited was 20 mg/cm^2^. Both cathode and anode were punched into a 15 mm diameter disk. The filter paper was punched into a 19 mm disk and used as the separator. Then, 0.3 mL of ZnSO_4_ (1 M) was added to the cell. The testing cells were fabricated as a coin cell (CR2032).

Electrochemical measurements were carried out using a CR2032 coin cell. Cyclic Voltammetry (CV) was performed by Potentiostat (VersaStat3, Princeton Applied Research) at a scan rate of 0.5 mV/s in the voltage range 1.0–1.8 V versus Zn^2+^/Zn. A battery testing system (BTS-5V10mA, Neware, China) was used to investigate the performance of the battery. The charge-transfer resistance, as illustrated by the Nyquist plots for the cathode, was carried out using an Electrochemical Impedance Spectroscopy (EIS) technique using an amplitude of 10 mV in the frequency range of 1–100,000 Hz.

## Results and discussion

**Figure 1:**
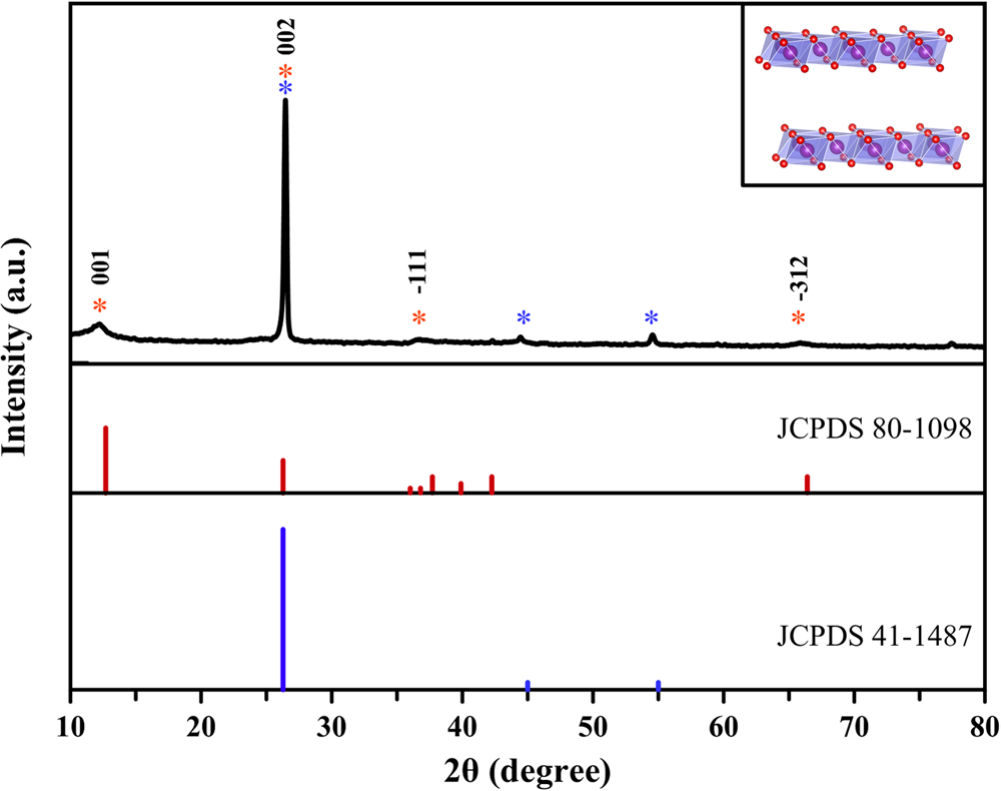
XRD pattern of the synthesized δ-MnO_2_ nanoflower/graphite (MNG) and crystallographic structure of δ-MnO_2_ (inset).

In this study, δ-MnO_2_ nanoflower/graphite (MNG) was prepared by a modified method of Liu *et al*.^22^. In Fig. 1, the XRD patterns of δ-MnO_2_ and graphite are shown. Thus, it can be seen that both patterns, i.e. δ-MnO_2_ (JSPDS card no. 80–1098)^26,27^ and graphite (JSPDS card no. 41–1487)^28^ match well. In the XRD pattern of MNG. The diffraction peaks (2θ) at 12.2°, 26.5°, 36.6°, and 65.6° correspond to the (001), (002), (−111) and (−312) crystal planes of δ-MnO_2_, respectively^29^. The high-intensity diffraction peak of MNG at 26.5° indicates the high crystallinity of graphite. δ-MnO_2_ possesses a planar layered-structure as illustrated by the inset of Fig. 1. This structure suggests that the interlayer gap is easy to insert/extract foreign cations and thereby can be useful for energy storage applications^27^. Further, the XRD analysis confirmed that the delta (δ) phase was presented in the pristine δ-MnO_2_ sample (see Supplementary Information Fig. S1).

**Figure 2:**
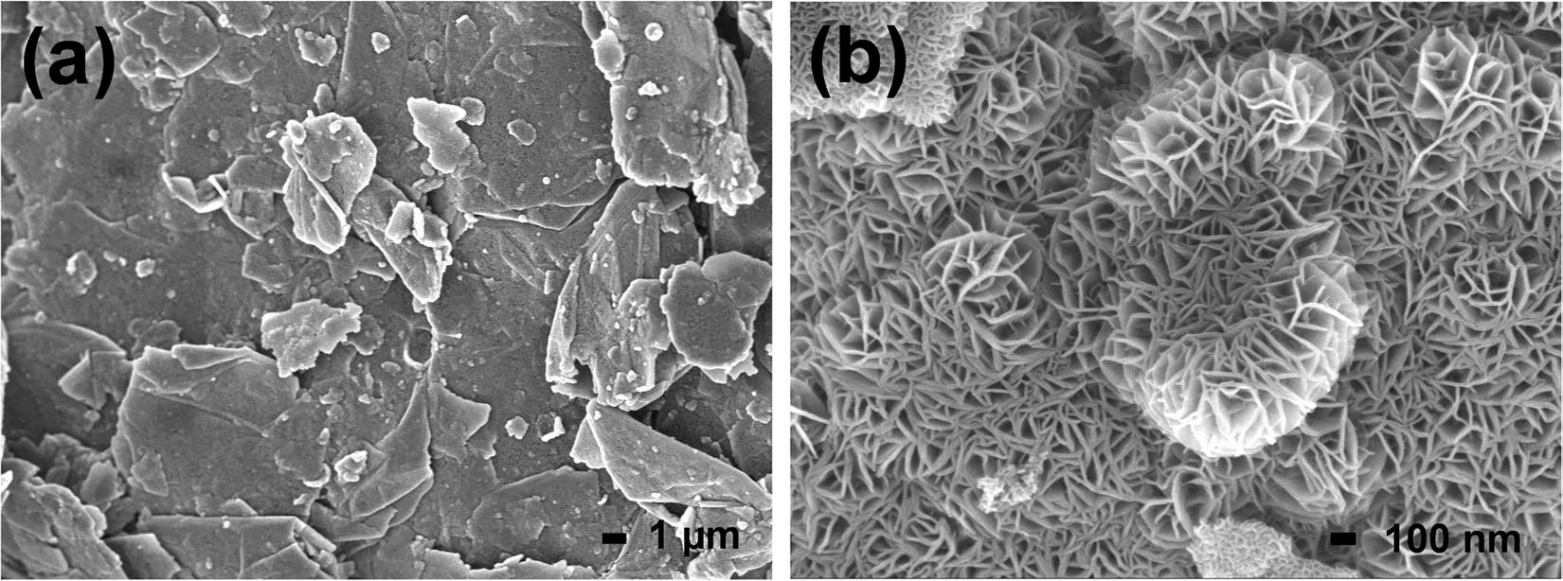
FESEM images of the synthesized δ-MnO_2_ nanoflower/graphite (MNG): (**a**) low magnification image, and (**b**) high–magnification image.

In Fig. 2(a), the FESEM image of MNG is shown. It is observed that the numerous MnO_2_ nanoflowers constructed on the graphite surface reveal a flake-like sample. Figure 2(b) shows the higher magnification image of MNG which indicates that many petals can interconnect forming micropores about 50 nm in diameter size. The MNG having micropores among petals will assist in increasing the contact area between the electrolyte and cathode material as well as ensure fast ion transfer in the charge/discharge process^22^.

**Figure 3:**
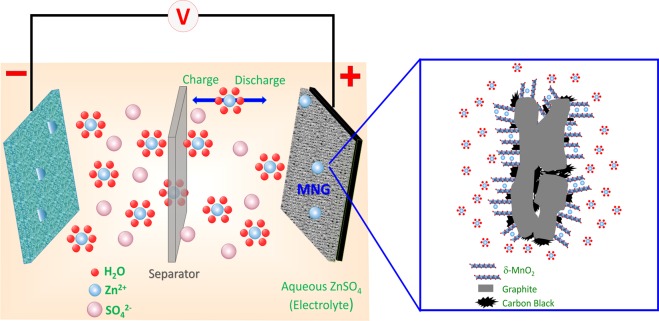
Schematics of the chemistry of the zinc-ion battery. Zn^2+^ ions migrate between tunnels of the MNG cathode and Zn anode. The inset on the right shows Zn^2+^ ion insertion and interconnection between δ-MnO_2_ and graphite.

In Fig. 3, the battery configuration in this study which is composed of the MNG cathode, zinc anode, and ZnSO_4_ aqueous electrolyte is shown. During discharging, anodic zinc is dissolved in the form of Zn^2+^ ions into an aqueous electrolyte, containing Zn^2+^ ions and rapidly solvate in the form of solvated Zn^2+^ ion. Then, they diffuse and pass through the separator to the MNG cathode. The solvated Zn^2+^ ions are de-solvated in the form of Zn^2+^ ions and intercalate into δ-MnO_2_ structure^30,31^ as illustrated by the inset of Fig. 3. Further, an electron current starts to flow in the electrical loop from the electrical conduction of graphite. These three processes can be reversed by (1) the de-intercalation of Zn^2+^ ions from MNG cathode; then (2) solvated species are formed and lastly (3) Zn^2+^ ions are reduced to Zn and deposited back on the zinc anode, respectively. The electrochemical reaction may be expressed as in Eq. (1) anode reaction and Eq. (2) cathode reaction:

1 $${\rm{Zn}}\iff {{\rm{Zn}}}^{2+}+2{{\rm{e}}}^{-}$$

2 $${{\rm{Zn}}}^{2+}+2{{\rm{e}}}^{-}+{{\rm{\delta }}-\mathrm{MnO}}_{2}\iff {{\rm{\delta }}-\mathrm{ZnMnO}}_{2}$$

During the electrochemical Zn^2+^ ion insertion^13^, the layered type δ-MnO_2_ structure can transform to spinel-type ZnMn_2_O_4_ with Mn(III) state and layered type δ-Zn_x_MnO_2_ with Mn(II) state.

**Figure 4:**
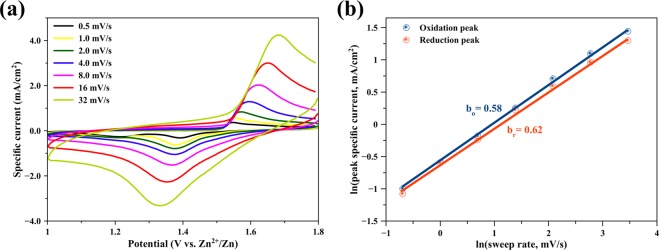
(**a**) Cyclic voltammograms of δ-MnO_2_ nanoflower/graphite (MNG) cycling at different sweep rates, and (**b**) the fitted lines: ln(peak specific current) versus ln(sweep rate).

To investigate the kinetics of the MNG electrode, cyclic voltammograms using sweep rates of 0.5 to 32 mV/s in the voltage range 1.0–1.8 V versus Zn^2+^/Zn were measured using CR2032 coin cells. As shown in Fig. 4(a), a dominating pair of redox peaks exhibits increasing currents when the sweep rates increase, which do not display rectangular-shape and symmetrical voltammograms even at high scan rates, compared to MnO_2_/activated carbon composite for supercapacitors^32^. It is noted that the MNG electrode does not present the capacitive behavior of the electrode^33,34^. The capacitive effect is characterized by analyzing the cyclic voltammetry data at different sweep rates as in Eq. (3):

3 $$i=a{v}^{b}$$

where *i* is the peak specific current, *v* is a potential sweep rate and *a, b* are adjustable parameters. The redox reaction is limited by the diffusion-controlled behavior; the peak current *i* varies as *v*^*1/2*^. Although the capacitance contribution suggests that the peak current *i* varies as *v*^35,36^. Equation (3) can be taken with logarithm and can be expressed according to Eq. (4):

4 $$\mathrm{ln}\,{i}={b}\,\mathrm{ln}\,{v}+\,\mathrm{ln}\,{a}$$

The *b* value denotes the slope of the plot of ln*i* versus ln*v*. When *b* value is close to 1, the system is mainly controlled by capacitance; when *b* value is close to 0.5, the Zn^2+^ ion insertion process dominates. Figure 4(b) shows the ln*i* versus ln*v* plots at oxidation and reduction process of the cyclic voltammogram. The *b*_*o*_ (oxidation process) and *b*_*r*_ (reduction process) of the MNG cathode are 0.58 and 0.62, respectively. Since the average *b* values are close to 0.5, it may imply that the redox reactions on the MNG cathode are controlled by the diffusion process. MnO_2_ is a transition metal oxide that typically displays the pseudocapacitance behavior^37^. The capacitive-controlled process occurs only on the surface. However, in the case of MNG cathode, the characteristic of Zn^2+^ ions insertion/extraction deviates from capacitive-controlled process towards the diffusion-controlled process. That is, the insertion/extraction of Zn^2+^ ions occur not only on the surface but also the pores inside. The result shows good agreement of a fast Zn^2+^ ion insertion/extraction or high rate property for the battery^35^.

**Figure 5:**
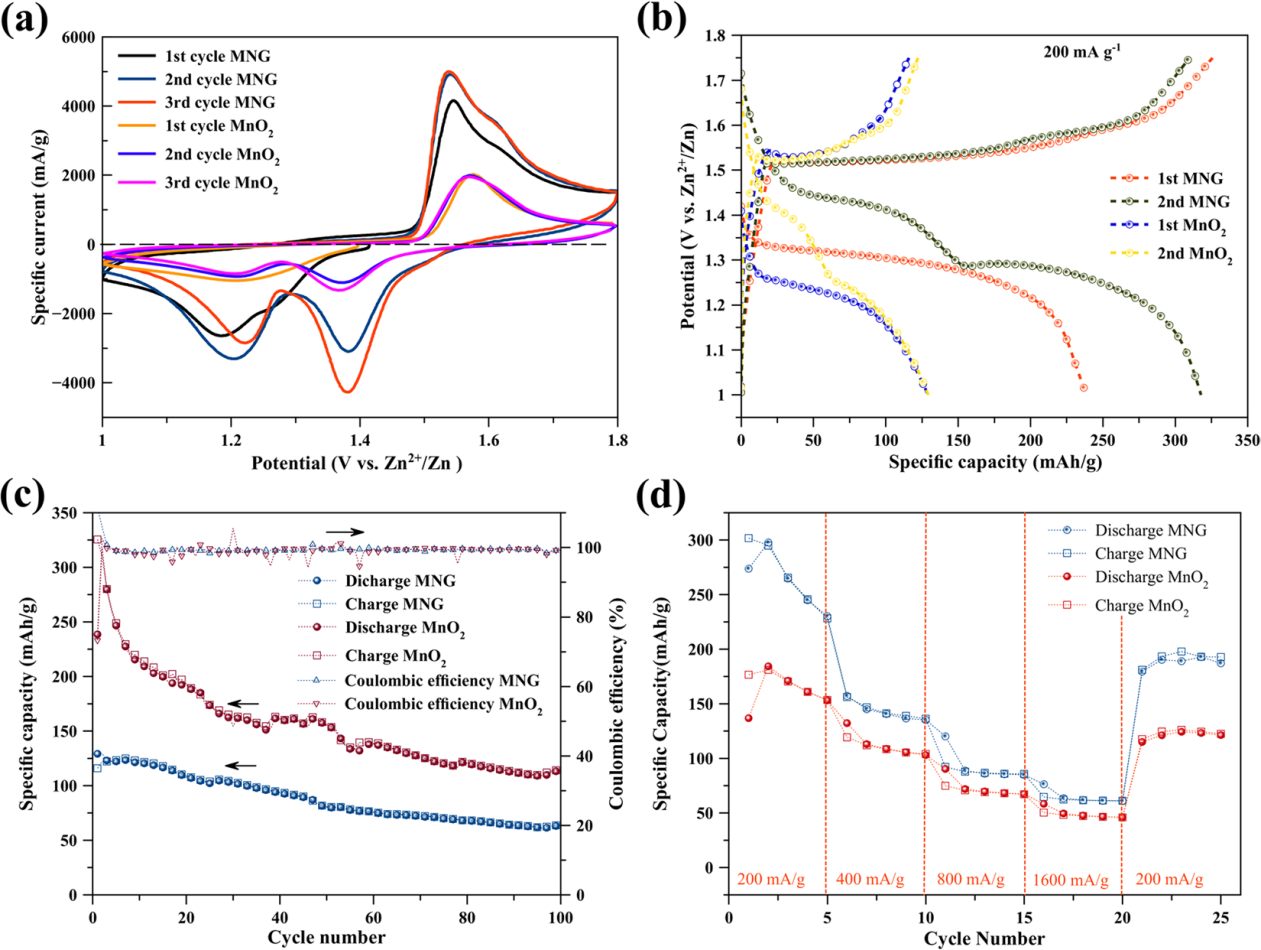
Performances of the batteries: (**a**) cyclic voltammograms of the batteries at a scan rate of 0.5 mV/s, (**b**) galvanostatic charge-discharge profile of the batteries at 200 mA/g, (**c**) cycling performance of the batteries at 400 mA/g, and (**d**) rate capability of the batteries at different discharge rates.

In order to compare the improved electrochemical properties of MNG as the cathode for ZIB, the pristine δ-MnO_2_ is used as a comparable cathode. Figure 5 (a) shows the CV profiles of the pristine δ-MnO_2_ and MNG electrodes at a scan rate of 0.5 mV/s over the potential range 1.0–1.8 V for the initial three cycles. During the first cycle, two distinct peaks are observed at 1.20 and 1.57 V for the pristine δ-MnO_2_ electrode and at 1.18 and 1.54 V for MNG. The peaks in the low potential region appear at 1.20 and 1.18 V which can be attributed to Zn^2+^ ion insertion into the δ-MnO_2_ host structure. In the higher potential region, two oxidation peaks can be seen at 1.57 and 1.54 V for δ-MnO_2_ and MNG, respectively, which correspond to the extraction of Zn^2+^ ions from the δ-MnO_2_ host structure. The results suggest that, the oxidation state of Mn is reduced to Mn^3+^ states and is oxidized back to Mn^4+^ states, during Zn^2+^ ion insertion and extraction, respectively^13,14,18,38^. On subsequent cycling, two distinct peaks appear at 1.37 and 1.21 V for Zn^2+^ ion insertion into δ-MnO_2_ whereas the peaks at 1.38 and 1.20 V were observed for MNG in the low voltage region. In the high voltage region, the peak at 1.57 and shoulder at 1.62 V can be clearly seen for the pristine δ-MnO_2_. Likewise, the peak at 1.53 V and shoulder at 1.60 V can be clearly seen for the MNG electrodes. The cyclic voltammogram, having two peaks during discharge and having a peak with shoulder during charge, exhibits typical characteristics of the electrochemical insertion/extraction of Zn^2+^ ions in MnO_2_ structure^12,13,39–41^. These results with two peaks during discharge may be more clearly described by the two-step reaction of Zn^2+^ ion insertion in electrochemical reaction^12,38^ and spinel-type ZnMn_2_O_4_ transformation^33^. In the following scan cycles, the peaks at 1.37 V for δ-MnO_2_ and at 1.38 for MNG increase gradually during discharge indicating an activation process^38^. The CV curve of MNG exhibits a higher peak intensity and a larger enclosed area when compared with the pristine δ-MnO_2_ indicating improved electrochemical performance and fast Zn^2+^ ion insertion/extraction^42^.

Figure 5(b) shows the first and second discharge/charge profiles of the pristine δ-MnO_2_ and MNG cathode in a coin cell battery when cycled at a specific current density of 200 mA/g in the potential range of 1.0–1.8 V. The battery fabricated with the pristine δ-MnO_2_ and MNG host cathode material under open air condition displays an open-circuit voltage (OCV) of about 1.4 V. The first discharge capacity for MNG is 235 mAh/g whereas the pristine δ-MnO_2_ registers only 130 mAh/g. Compared to todorokite-type MnO_2_^14^ and δ-MnO_2_ nano-flake^13^, the initial discharge capacity can deliver only 98 mAh/g (at 50 mA/g) and 122 mAh/g (at 83 mA/g), respectively. It is clear that δ-MnO_2_ supported on graphite samples can accommodate more numbers of Zn^2+^ ions than the unsupported δ-MnO_2_ (pristine δ-MnO_2_). It appears that the nanoflower δ-MnO_2_ in the structure of MNG tend to enhance the electrode/electrolyte contact area, thereby favoring Zn^2+^ ion insertion^41^. In addition, MNG shows a longer horizontal discharge curve than that of δ-MnO_2_, suggesting a more stable Zn^2+^ ion insertion into the MNG than in the pristine δ-MnO_2_. On the subsequent cycle, during the continuous discharge, the voltage profiles present two distinct plateaus at 1.45 and 1.25 V for MNG and 1.4 and 1.2 V for δ-MnO_2_. These characteristics were also observed for MnO_2_ electrodes in aqueous ZIB systems^14,43^. It can be implied that Zn^2+^ ions can insert into the layered δ-MnO_2_^39^ which is in agreement with the two distinct peaks during discharge, as shown in Fig. 5(a).

Figure 5(c) displays the cycling behavior and corresponding coulombic efficiency of the pristine δ-MnO_2_ and MNG electrodes, under the specific current density of 400 mA/g. At the 100^th^ cycle, the discharge and charge capacities registered by δ-MnO_2_ were 63.3 and 63.8 mAh/g, respectively while that of MNG were 113.4 and 114.2 mAh/g, respectively. The lower capacity retention of δ-MnO_2_ may result from low intrinsic electronic conductivity because of the appearance of unstable Mn^3+^ states during zinc-ion insertion^10^. The higher capacity retention of MNG may indicate that the electronic conductivity of MNG is improved. In the initial cycles, gradual capacity fade was observed for the pristine δ-MnO_2_ electrode, but fast capacity fade was observed for the MNG electrode. The formation of ZnMn_2_O_4_ with the Jahn-Teller Mn^3+^ ion may contribute to electrode degradation and hence lead to the fast capacity fade^13^. However, MNG demonstrates higher capacity than the pristine δ-MnO_2_. Over long-term cycling, the coulombic efficiency of both host material electrodes is maintained at around 100%. Thus, this clearly demonstrates that no irreversible capacity losses occurred^41^.

In Fig. 5(d), the rate performances of the pristine δ-MnO_2_ and MNG host material cathodes are shown. Cycling takes place at various specific current densities of 200, 400, 800 and 1600 mA/g, namely 5 times for each rate. The rate performance of MNG is significantly higher than those of the pristine δ-MnO_2_. It is indicated that nanoscale morphology of δ-MnO_2_ nanoflowers on graphite increases the contact area between the electrode and the electrolyte and provides more electrochemically active sites for ion-insertion^13^. Graphite not only improves the electronic conductivity of the MNG electrode but also tends to disperse the δ-MnO_2_ nanoflower sites. The MNG cathode can be charged and discharged at different rates; a high rate of 1600 mA/g leads to a discharge and charge capacity of 76 and 64 mAh/g, respectively. When cycled at a specific current density of 200 mA/g, the MNG cathode can deliver a discharge and charge capacity of 181 and 179 mAh/g, respectively. This behavior indicates that the MNG cathode can well be considered for the Zn^2+^ ion storage material^10^. It is clear therefore that MNG can improve not only the cycling performance but also the rate performance for ZIBs.

**Figure 6:**
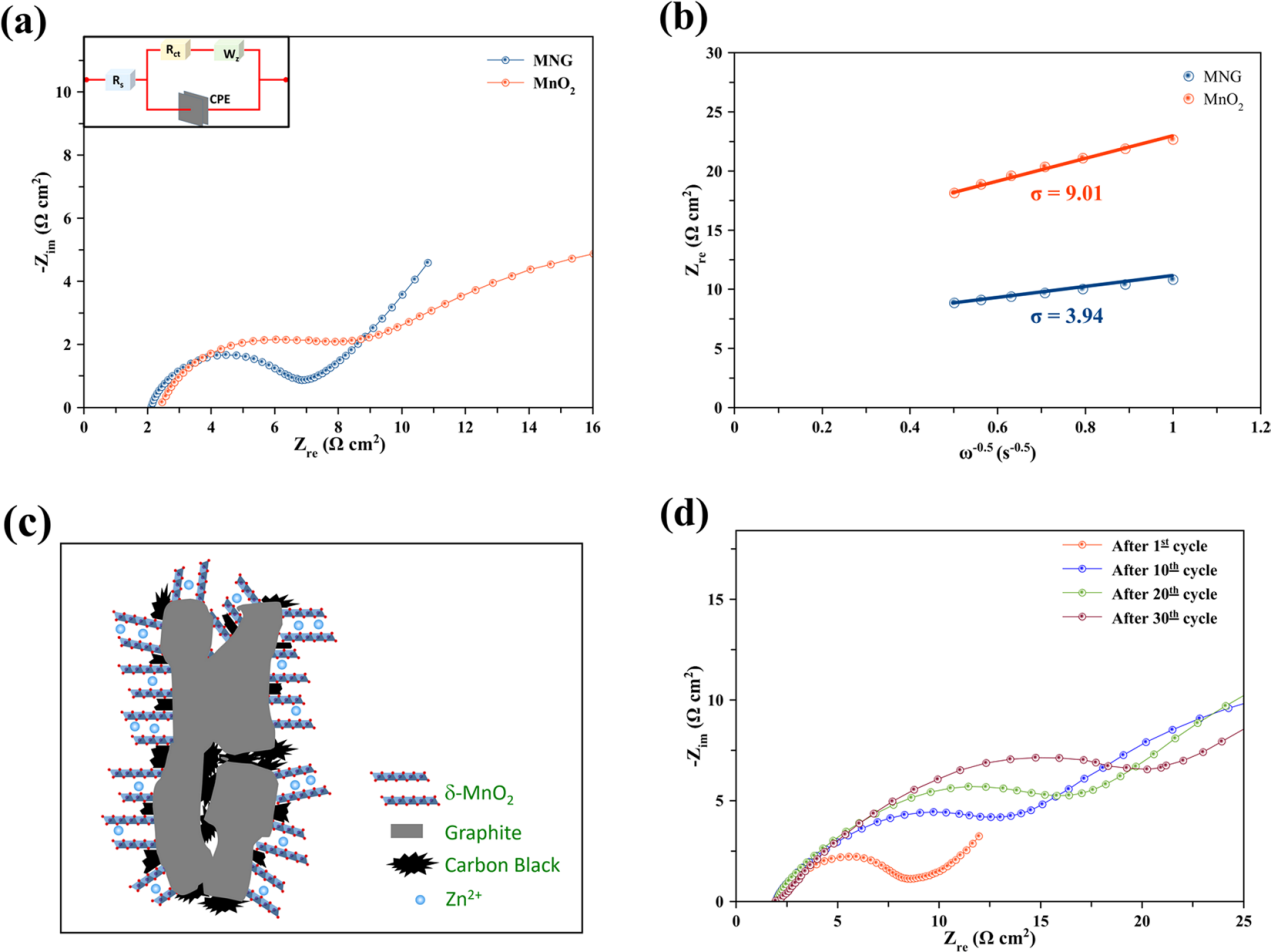
EIS results of the batteries: (**a**) Nyquist plot of EIS spectra, (**b**) relationship between real part of impedance versus ω^−0.5^ for the batteries using MNG and the pristine δ-MnO_2_, (**c**) schematic illustration for the Zn^2+^ ion insertion into the MNG electrode, and (**d**) Nyquist plot of EIS spectra of MNG at various charge/discharge cycles.

As displayed in Fig. 6(a), the difference in electrochemical conductivity before cycling between the pristine δ-MnO_2_ and MNG host cathodes was examined using EIS. The curves of the pristine δ-MnO_2_ and MNG host cathodes consist of depressed semicircles and diffusion drift which can be perfectly fitted using Randles equivalent circuit (see Inset Fig. 6(a)). Hence, in the equivalent circuit, R_s_ is the solution resistance, R_ct_ is the charge-transfer resistance at the interfaces and Z_w_ is the Warburg impedance related to the diffusion of Zn^2+^ ions. The R_ct_ value for the pristine δ-MnO_2_ is 5.9 Ω and the value reduces to 4.8 Ω when supported on graphite (MNG). The result indicates that the electrical conductivity of the MNG sample is improved by constructing a conductive support using the graphite. The relationship between real impedance (Z′) and angular frequency (ω) in the low frequency region can be expressed accordingly by Eq. (5)^44^:

5 $${Z}^{\text{'}}={R}_{s}+{R}_{ct}+\sigma {\omega }^{-0.5}$$

where σ is the Warburg factor which is relative to Z′- ω obtained from the slope of the lines in Fig. 6(b). The diffusion coefficient of zinc ion can be calculated as in Eq. (6)^45^:

6 $$D={R}^{2}{T}^{2}/2{A}^{2}{n}^{4}{F}^{4}{C}^{2}{\sigma }^{2}$$

where *R* is the gas constant, *T* is the absolute temperature, *n* is the number of electrons per molecule oxidized, *A* is the surface area, *F* is Faraday’s constant, *C* is the concentration and *D* is the diffusion coefficient.

As shown in Fig. 6(b), the slope which is the σ value of MNG (3.94) host electrode is lower than that of the pristine δ-MnO_2_ (9.01) indicating that the diffusion coefficient of MNG is higher than that of the pristine δ-MnO_2_, as in Eq. (6). It is clear that the MNG host material electrode can enhance the diffusion coefficient of Zn^2+^ ion, highlighting the electrical conductivity improvement^44,46^.

In Fig. 6(c), the MNG cathode which is a δ-MnO_2_ structure supported on graphite is drawn and illustrated. It is highly possible that Zn^2+^ ions can insert into the δ-MnO_2_ nanoflower structure having short diffusion length. Thereby, electrochemical performance can be improved during cycling at high rate^47^.

In Fig. 6(d), after charge/discharge cycling, the EIS measurements of MNG are shown. After the 1^st^, 10^th^, 20^th^, and 30^th^ cycles, the MNG host cathode exhibits the R_ct_ values, namely 6.51, 10.9, 13.7 and 18.2 Ω, respectively. After the first cycle, the charge-transfer resistance increases which indicates that the intercalation of Zn^2+^ ions into the δ-MnO_2_ structure becomes more difficult. The conduction of ions before intercalation depends not only on the cathode material but also on the electrolyte access into the cathode. The porosity of the cathode material is an important factor which can affect the electrolyte access. The SEM image of MNG and the pristine δ-MnO_2_ compound is displayed in the Supplementary Information Fig. S2.

## Conclusion

In summary, δ-MnO_2_ nanoflower supported on graphite flake was synthesized and used as an intercalation host material for a rechargeable ZIB. While the XRD studies confirmed the crystallographic structure, the FESEM studies revealed that the sample showed a nanoflower-like morphology having micropores about 50 nm in diameter. This study demonstrates that a diffusion process controlled the electrochemical reactions of the MNG cathode. When tested in ZIB, the MNG sample registered a prominent discharge capacity of 235 mAh/g, which was higher than the discharge capacity of pristine δ-MnO_2_ (130 mAh/g). After the 100^th^ cycle, the discharge capacity registered by the δ-MnO_2_ was only 63.3 mAh/g whereas the MNG delivered a discharge capacity of 113.4 mAh/g. Moreover, the conductivity of the supporting graphite improved the conductivity by reducing the charge transfer resistance of the cathode materials. The present results concluded that the supporting graphite not only improved the electrical conductivity but also enhanced the specific capacity and the cycling performance of the pristine δ-MnO_2_. Thus, MNG is one of promising candidate cathode materials for ZIBs.

## Supplementary information

Supplementary Information

## Data Availability

The authors declare that all relevant data are within the paper Competing interests: The authors declare no potential conflict of interest.

## References

[1] Misnon II (2014). High performance MnO_2_ nanoflower electrode and the relationship between solvated ion size and specific capacitance in highly conductive electrolytes. Mater. Res. Bull..

[2] Lao-atiman W, Julaphatachote T, Boonmongkolras P, Kheawhom S (2017). Printed transparent thin film Zn-MnO_2_ battery. J. Electrochem. Soc..

[3] Suren S, Kheawhom S (2016). Development of a high energy density flexible zinc-air battery. J. Electrochem. Soc..

[4] Hosseini S (2018). Discharge Performance of Zinc-Air Flow Batteries Under the Effects of Sodium Dodecyl Sulfate and Pluronic F-127. Sci. Rep..

[5] Wang J-W, Chen Y, Chen B-Z (2016). Synthesis and control of high-performance MnO_2_/carbon nanotubes nanocomposites for supercapacitors. J. Alloy Compd..

[6] Hosseini S, Han SJ, Arpornwichanop A, Yonezawa T, Kheawhom S (2018). Ethanol as an electrolyte additive for alkaline zinc-air flow batteries. Sci. Rep..

[7] Lao-atiman, W. *et al*. Model-Based Analysis of an Integrated Zinc-Air Flow Battery/Zinc Electrolyzer System. *Frontiers in Energy Research* **7**, 10.3389/fenrg.2019.00015 (2019).

[8] Chen J (2014). Electrochemical properties of MnO_2_ nanorods as anode materials for lithium ion batteries. Electrochim. Acta.

[9] Kim J-S (2015). High-capacity nanostructured manganese dioxide cathode for rechargeable magnesium ion batteries. J. Power Sources.

[10] Alfaruqi MH (2016). A high surface area tunnel-type α-MnO_2_ nanorod cathode by a simple solvent-free synthesis for rechargeable aqueous zinc-ion batteries. Chem. Phys. Lett..

[11] Kao-ian W, Pornprasertsuk R, Thamyongkit P, Maiyalagan T, Kheawhom S (2019). Rechargeable Zinc-Ion Battery Based on Choline Chloride-Urea Deep Eutectic Solvent. Journal of The Electrochemical Society.

[12] Alfaruqi MH (2015). Enhanced reversible divalent zinc storage in a structurally stable α-MnO_2_ nanorod electrode. J. Power Sources.

[13] Alfaruqi MH (2015). A layered δ-MnO_2_ nanoflake cathode with high zinc-storage capacities for eco-friendly battery applications. Electrochem commun..

[14] Lee J, Ju JB, Cho WI, Cho BW, Oh SH (2013). Todorokite-type MnO_2_ as a zinc-ion intercalating material. Electrochim. Acta.

[15] Toupin M, Brousse T, Bélanger D (2004). Charge storage mechanism of MnO_2_ electrode used in aqueous electrochemical capacitor. Chem. Mater..

[16] Feng M (2017). Manganese oxide electrode with excellent electrochemical performance for sodium ion batteries by pre-intercalation of K and Na ions. Sci. Rep..

[17] Song J, Kim J, Kang T, Kim D (2017). Design of a porous cathode for ultrahigh performance of a Li-ion battery: An overlooked pore distribution. Sci. Rep..

[18] Wei C, Xu C, Li B, Du H, Kang F (2012). Preparation and characterization of manganese dioxides with nano-sized tunnel structures for zinc ion storage. J. Phys. Chem. Solids.

[19] Renuka R, Ramamurthy S (2000). An investigation on layered birnessite type manganese oxides for battery applications. J. Power Sources.

[20] Zhu C (2011). One-step electrochemical approach to the synthesis of Graphene/MnO_2_ nanowall hybrids. Nano Res..

[21] Zhou J (2013). Novel synthesis of birnessite-type MnO_2_ nanostructure for water treatment and electrochemical capacitor. Ind. Eng. Chem. Res..

[22] Liu J (2015). *In situ* chemical synthesis of sandwich-structured MnO_2_/graphene nanoflowers and their supercapacitive behavior. Electrochim. Acta.

[23] Phillips C, Al-Ahmadi A, Potts S-J, Claypole T, Deganello D (2017). The effect of graphite and carbon black ratios on conductive ink performance. J. Mater. Sci..

[24] Wongrujipairoj Krittaporn, Poolnapol Laksanaporn, Arpornwichanop Amornchai, Suren Sira, Kheawhom Soorathep (2016). Suppression of zinc anode corrosion for printed flexible zinc-air battery. physica status solidi (b).

[25] Chernozatonskii LA, Sorokin PB, Belova EÉ, Brüning J, Fedorov AS (2006). Metal-semiconductor (semimetal) superlattices on a graphite sheet with vacancies. JETP Lett..

[26] Li F (2015). MnO_2_ nanostructures with three-dimensional (3D) morphology replicated from diatoms for high-performance supercapacitors. J. Mater. Chem. A.

[27] Thapa AK (2014). Synthesis of mesoporous birnessite-MnO_2_ composite as a cathode electrode for lithium battery. Electrochim. Acta.

[28] Rajarao R, Bhat BR (2012). Large scale synthesis of carbon nanofibres on sodium chloride support. Nanomater. Nanotechno..

[29] Zhang J, Li Y, Wang L, Zhang C, He H (2015). Catalytic oxidation of formaldehyde over manganese oxides with different crystal structures. Catal. Sci. Technol..

[30] Kundu D (2018). Aqueous vs. nonaqueous Zn-ion batteries: consequences of the desolvation penalty at the interface. Energy Environ. Sci..

[31] Hayes AC, Kruus P, Adams WA (1984). Raman spectroscopic study of aqueous (NH_4_)_2_SO_4_ and ZnSO_4_ solutions. J. Solution Chem..

[32] Wang J-W, Chen Y, Chen B-Z (2015). A synthesis method of MnO_2_/activated carbon composite for electrochemical supercapacitors. J. Electrochem. Soc..

[33] Ji C, Ren H, Yang S (2015). Control of manganese dioxide crystallographic structure in the redox reaction between graphene and permanganate ions and their electrochemical performance. RSC Adv..

[34] Yang Y-j (2009). Nanostructured MnO_2_/exfoliated graphite composite electrode as supercapacitors. J. Alloy Compd..

[35] Li J (2017). Improved Li-ion diffusion process in TiO_2_/rGO anode for lithium-ion battery. J. Alloy Compd..

[36] Simon P, Gogotsi Y, Dunn B (2014). Where do batteries end and supercapacitors begin. Science.

[37] Chen D (2015). Probing the charge storage mechanism of a pseudocapacitive MnO_2_ electrode using in operando raman spectroscopy. Chem. Mater..

[38] Guo Xiaotong, Li Jianming, Jin Xu, Han Yehu, Lin Yue, Lei Zhanwu, Wang Shiyang, Qin Lianjie, Jiao Shuhong, Cao Ruiguo (2018). A Hollow-Structured Manganese Oxide Cathode for Stable Zn-MnO2 Batteries. Nanomaterials.

[39] Alfaruqi MH (2018). Structural transformation and electrochemical study of layered MnO_2_ in rechargeable aqueous zinc-ion battery. Electrochim. Acta.

[40] Qiu W (2017). High-performance flexible quasi-solid-state Zn–MnO_2_ battery based on MnO_2_ nanorod arrays coated 3D porous nitrogen-doped carbon cloth. J. Mater. Chem. A.

[41] Alfaruqi MH (2015). Electrochemically induced structural transformation in a γ-MnO_2_ cathode of a high capacity zinc-Ion battery system. Chem. Mater..

[42] Islam S (2017). Carbon-coated manganese dioxide nanoparticles and their enhanced electrochemical properties for zinc-ion battery applications. J. Energy Chem..

[43] Xu C, Li B, Du H, Kang F (2012). Energetic zinc ion chemistry: the rechargeable zinc ion battery. Angew. Chem. Int. Ed. Engl..

[44] Liu H (2006). Kinetic study on LiFePO_4_/C nanocomposites synthesized by solid state technique. J. Power Sources.

[45] Bard, A. J. & Faulkner, L. R. *Electrochemical Methods*. 2 edn, 211 (JOHN WILEY & SONS, INC. 2001).

[46] Li H (2019). Dual-carbon confined SnO_2_ as ultralong-life anode for Li-ion batteries. Ceram. Int..

[47] Cao Q (2007). A novel carbon-coated LiCoO_2_ as cathode material for lithium ion battery. Electrochem commun..

