# Supplementary material for: δ-MnO2 nanoflower/graphite cathode for rechargeable aqueous zinc ion batteries
Source: Sci Rep. 2019 Jun 11;9:8441. doi: 10.1038/s41598-019-44915-8 (PMC6560026; doi:10.1038/s41598-019-44915-8)
Supplement: Supplementary file 1 — Supplementary Information [file 41598_2019_44915_MOESM1_ESM.docx]

**δ-MnO_2_ nanoflower/graphite cathode for rechargeable aqueous zinc ion batteries**

Sonti Khamsanga^1^, Rojana Pornprasertsuk^2,3,4^, Tetsu Yonezawa^5^, Ahmad Azmin Mohamad^6^, Soorathep Kheawhom^1,3,*^

^1^Department of Chemical Engineering, Faculty of Engineering, Chulalongkorn University, Bangkok, 10330, Thailand.

^2^Department of Materials Science, Faculty of Science, Chulalongkorn University, Bangkok, 10330, Thailand.

^3^Research Unit of Advanced Materials for Energy Storage, Chulalongkorn University, Bangkok, 10330, Thailand.

^4^Center of Excellence in Petrochemical and Materials Technology, Chulalongkorn University, Bangkok, 10330, Thailand.

^5^Division of Materials Science and Engineering, Faculty of Engineering, Hokkaido University, Kita 13 Nishi 8, Sapporo, Hokkaido, 060-8628, Japan.

^6^School of Materials and Mineral Resources Engineering, Universiti of Sains Malaysia, 14300 Nibong Tebal, Pulau Pinang, Malaysia.

*soorathep.k@chula.ac.th


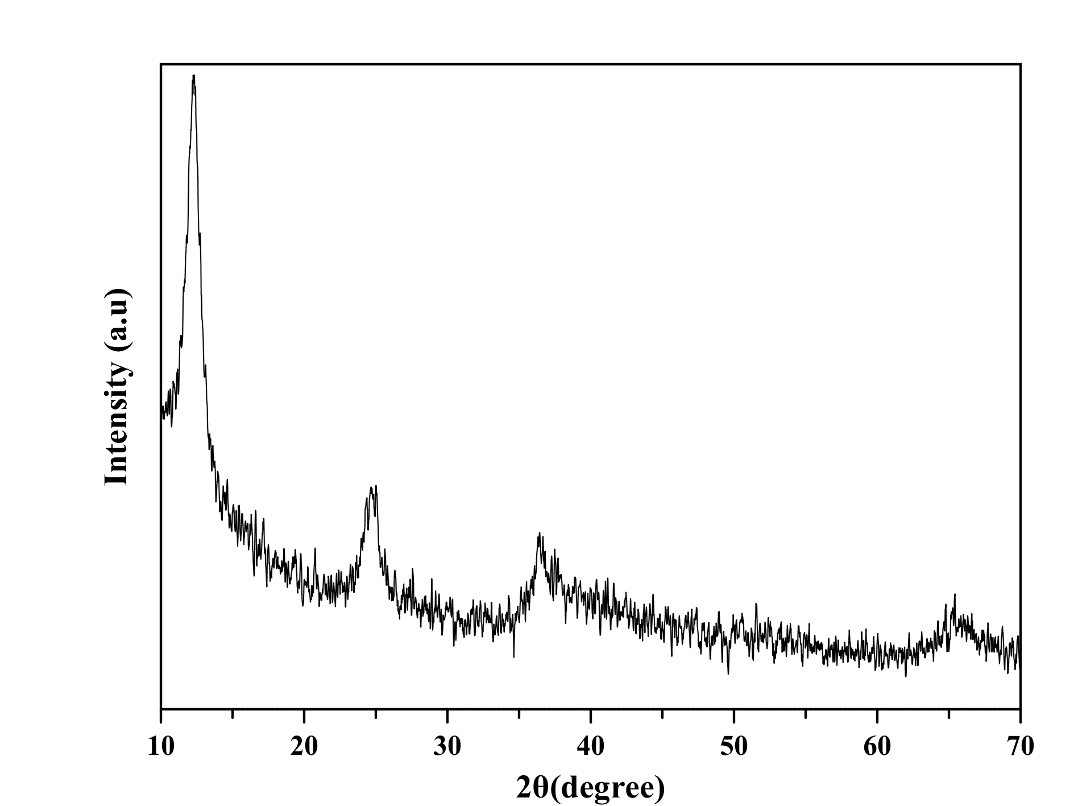
**Supplementary Information**

**Figure S1.** XRD pattern and crystallographic structure of the pristine δ-MnO_2_.


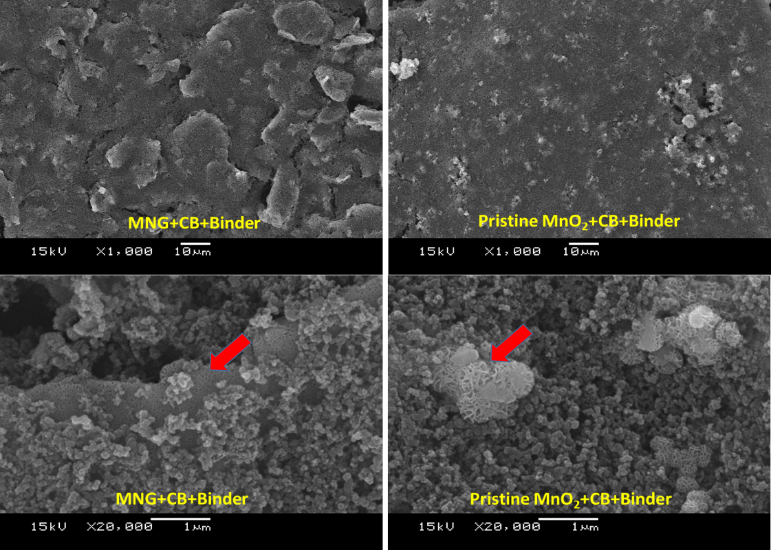


**(a)**

**(b)**

**Figure S2.** SEM images of (a) MNG, and (b) the pristine MnO_2_ compound at different magnification.
